# Long-term safety and effectiveness of lurasidone in adolescents and young adults with schizophrenia: pooled post hoc analyses of two 12-month extension studies

**DOI:** 10.1186/s12991-024-00502-4

**Published:** 2024-07-18

**Authors:** Fabrizio Calisti, Michael Tocco, Yongcai Mao, Robert Goldman

**Affiliations:** 1 Angelini Pharma S.P.A, Viale Amelia, 70, 00181 Rome, Italy; 2 Former Employee of Sumitomo Pharma America, Inc, 84 Waterford Drive, Marlborough, MA 01752 USA; 3 Sumitomo Pharma America, Inc, 84 Waterford Drive, Marlborough, MA 01752 USA

**Keywords:** Schizophrenia, Atypical antipsychotics, Lurasidone, Long-term

## Abstract

**Background and Objectives:**

The aim of this analysis was to evaluate the long-term safety and effectiveness of lurasidone in the treatment of schizophrenia in adolescents and young adults (13–25).

**Methods:**

The 2 pooled studies used similar designs and outcome measures. Patients (13–25) with schizophrenia completed an initial double-blind 6-week trial of lurasidone (40 and 80 mg/day) in the adolescent trial and (80 and 160 mg/day) in the young adult trial. In open-label long-term trials, adolescent patients were treated with 20–80 mg/day lurasidone, and adults were treated with 40–160 mg/day lurasidone. Efficacy was evaluated based on the Positive and Negative Syndrome Scale (PANSS) and Clinical Global Impression-Severity Scale (CGI-S).

**Results:**

The safety population consisted of 306 patients (mean age, 16.2 years; 208 patients (68.0%) who completed 12 months of treatment; 8.2% who discontinued treatment by 12 months due to an adverse event). The mean (SD) changes in the PANSS total score from the extension baseline to months 6 and 12 were − 11.8 (13.9) and – 15.3 (15.0), respectively (OC), and the mean (SD) changes in the CGI-S score were − 0.8 (1.0) and − 1.0 (1.1), respectively (OC). The most frequent adverse events were headache (17.6%), anxiety (11.4%), schizophrenia (9.8%), and nausea (9.8%). No clinically meaningful changes were observed in weight, metabolic parameters, or prolactin.

**Conclusions:**

In adolescents and young adults with schizophrenia, treatment with lurasidone was generally well tolerated and effective. Long-term treatment was associated with a continued reduction in symptoms of schizophrenia. Long-term treatment was associated with minimal effects on weight, metabolic parameters, and prolactin.

*Clinicaltrials.gov identifiers* D1050234, D1050302.

Schizophrenia is a chronic and severe illness with a peak age at onset of approximately 22 years and a median age at onset of 25 years [1]. Studies suggest that individuals with earlier-onset schizophrenia experience greater illness severity (including higher rates of comorbid psychiatric and substance abuse disorders), greater functional impairment, and poorer long-term outcomes than those with later-onset schizophrenia [2, 3]. Schizophrenia is ranked as one of the top five disorders in terms of disability-adjusted life years (DALYs) [4] a status that is largely attributable to the early onset of the illness, and its chronicity.

Due to its chronic, recurrent course of illness, clinical practice guidelines recommend maintenance treatment for individuals with a diagnosis of schizophrenia [5, 6]. Treatment with selected atypical antipsychotics and low-potency first-generation antipsychotics has been associated with weight gain and adverse metabolic effects. This is especially a problem in the treatment of younger patients and, over the course of long-term treatment, appears to contribute to the increased cardiovascular morbidity and mortality observed in patients with schizophrenia [7–15]. There is growing evidence that schizophrenia, type 2 diabetes and metabolic syndrome may exhibit shared genetic risk factors and that selected antipsychotics may increase this vulnerability, for example, by reducing the expression of the glucagon-like peptide-1 (GLP-1) receptor [16, 17].

Lurasidone is an atypical antipsychotic that has demonstrated short- and long-term efficacy in the treatment of schizophrenia in both adolescents [18, 19] and adults [20–27]. To date, few published studies have evaluated the efficacy and safety of atypical antipsychotics, specifically lurasidone, in patients who are 13–25 years old, a cohort that lies below the median age of onset of the disorder and is considered to have an early-onset phenotype [28]. The aim of this pooled post hoc analysis was to evaluate the efficacy and safety of 12 months of treatment with lurasidone after the completion of 6 weeks of treatment for acute exacerbation of schizophrenia in adolescents and young adults. We were especially interested in the ability of lurasidone to maintain the improvement that was achieved after the initial 6 weeks of treatment, the extent to which additional improvement occurred during long-term treatment, and whether maintenance therapy with lurasidone was associated with adverse weight and metabolic effects in this younger, at-risk population.

## Methods

Individual patient data reported in these post hoc analyses were pooled from 2 long-term studies that utilized very similar study designs and outcome measures [19, 26]. All patients were young, aged 13–25 years, and had completed one of two randomized, double-blind, placebo-controlled, 6-week studies of lurasidone for the treatment of an acute exacerbation of schizophrenia in adolescents [18] and in adults [29]. Details of the study design and entry criteria for the initial double-blind phase of each acute study are summarized in the primary reports. Briefly, in an adolescent study, [18] outpatients experiencing an acute exacerbation of schizophrenia were eligible for enrollment if they had a Clinical Global Impression, Severity (CGI-S) score ≥ 4 (moderate or greater) and a Positive and Negative Syndrome Scale (PANSS) total score ≥ 70. In the adult study, [29] hospitalized patients who experienced an acute exacerbation of schizophrenia were eligible for enrollment if they had a CGI-S score ≥ 4 and a PANSS total score ≥ 80. Key exclusion criteria were similar for both studies and included an acute or unstable medical condition: evidence of any other chronic disease of the central nervous system; alcohol or other drug abuse/dependence within the past 3–6 months; evidence of a severe, chronic movement disorder; or imminent risk of suicide (as judged by the study investigator). Both studies were approved by an institutional review board/ethics committee at each investigational site and both studies conducted in accordance with the International Conference on Harmonization Good Clinical Practice guidelines and with the ethical principles of the Declaration of Helsinki. After a full explanation of the study was provided, written informed consent was obtained from a parent or legal guardian and assent was obtained from each patient.

Patients who completed the 6-week double-blind phase of each study were invited to participate in the extension phase. If they agreed, and prior to the conduct of any study procedures, written informed consent was obtained from the young adults; for adolescents, written informed consent was obtained from a parent or legal guardian, and assent was obtained from each adolescent patient. This extension study was approved by an institutional review board/ethics committee at each investigational site and was conducted in accordance with the International Conference on Harmonization Good Clinical Practice guidelines and with the ethical principles of the Declaration of Helsinki.

Patients then continued treatment in two open-label long-term trials: adolescent patients were treated with 20–80 mg/day lurasidone, and adults were treated with 40–160 mg/day lurasidone. All adult and adolescent patients were started on a dose of 40 mg/day for 1 week in the extension study, regardless of their treatment group assignment in the original double-blind study. The dose of lurasidone could be adjusted at regularly scheduled visits within the flexible dose range. Lurasidone was taken orally, once daily in the evening with a meal or within 30 min after eating. As-needed concomitant treatment was permitted with lorazepam for intolerable anxiety/agitation (≤ 6 mg/day or equivalent dose of an alternative benzodiazepine), benzatropine (≤ 6 mg/day; or alternative anticholinergic medication) for movement disorders, and propranolol (≤ 120 mg/day) for akathisia. As-needed benzodiazepine or nonbenzodiazepine sedative-hypnotic agents were also permitted for insomnia.

The primary efficacy measure was the PANSS total score. Secondary efficacy outcomes included the CGI-S and the PANSS positive, negative, and general psychopathology subscales. Safety evaluations included assessments of adverse events, weight, and laboratory measures, including cholesterol, triglycerides, glucose, HbA1c, and prolactin. Movement disorders were assessed by the Simpson–Angus Scale, Barnes–Akathisia Rating Scale, and the Abnormal Involuntary Movement Scale [30–32].

### Statistical analysis

The pooled safety population consisted of all patients who completed the core acute-phase trial, continued into the current extension study, and received at least one dose of lurasidone in the extension phase of the study. All safety and effectiveness summaries were based on the safety population. No inferential statistics were calculated. For continuous variables (including both safety and effectiveness variables), descriptive summary statistics (N, mean, median, 95% confidence interval [CI], etc.) were reported at the core baseline, extension baseline, each post-extension baseline visit, week 52 endpoint, and endpoint in the extension study. In addition, changes from the core baseline and extension baseline were also reported in a similar way using summary statistics as described above. For treatment-emergent adverse events, the number and percentage of subjects with one or more events were summarized for overall incidence and discontinuations due to adverse events. Kaplan Meier analysis was used to estimate the median time to discontinuation during the extension phase for both the core lurasidone group and the placebo group.

Descriptive statistics were calculated using both observed case (OC) and last observation carried forward to endpoint (LOCF-endpoint) methods for changes in primary and secondary efficacy measures, including means, standard deviations, and 95% confidence intervals (CIs).

Change scores for primary and secondary efficacy measures were evaluated using mixed-model repeated-measures (MMRM) analysis. Responder and remission rates were analyzed using a logistic model. The response criterion for a ≥ 30% reduction in the PANSS total score was a double-blind baseline. Remission rates were evaluated using the Remission in Schizophrenia Working Group (RSWG) criteria for symptomatic remission, which require that a threshold of improvement be maintained for at least 6 months, [33] with symptom scores ≤ 3 on the following PANSS items: P1 (delusions), P2 (conceptual disorganization), P3 (hallucinatory behavior), G5 (mannerisms/posturing), G9 (unusual thought content), N1 (blunted affect), N4 (social withdrawal), and N6 (lack of spontaneity). [33]. A Kaplan Meier analysis was used to estimate the probability of relapse in patients who met the responder criteria at Month 3. A second Kaplan Meier relapse analysis estimated the probability of relapse in patients who met the criteria for sustained remission at Month 6. Relapse of psychotic symptoms was defined as the earliest occurrence of any of the following 4 criteria: (1) worsening of ≥ 30% in the PANSS total score from extension phase baseline; (2) rehospitalization for worsening of psychosis; or (3) emergence of suicidal ideation, homicidal ideation and/or risk of harm.

## Results

A total of 425 adolescents and young adults (13–25 years) completed the 6-week, double-blind, placebo-controlled trial, of whom 306 patients (72%) provided informed consent and continued the 12-month extension study, including 208 patients who were initially randomized to double-blind lurasidone (who continued on lurasidone in the extension study) and 98 patients who were randomized to double-blind placebo (who were switched to lurasidone at entry into the extension study). Switching was accomplished while maintaining the double-blind nature of the initial 6-week trial.

Demographic and clinical characteristics at the extension phase baseline were similar for patients receiving lurasidone or placebo during the initial core study, except for greater severity in the core placebo group according to the PANSS total score (79.2 vs. 72.4) and CGI-Severity score (4.2 vs. 3.7; Table 1).

**Table 1:** Baseline characteristics by treatment assignment during the initial randomized, double-blind, 6-week study (safety population)

	Lurasidone → Lurasidone (N = 208)	Placebo → Lurasidone (N = 98)
Male, n (%)	135 (64.9)	61 (62.2)
Age, years, mean (SD)	16.3 (2.3)	16.0 (2.5)
Race, %		
White	68.8	68.4
Black/African-American	11.5	16.3
Asian	10.1	7.1
Other	9.6	8.2
Age at onset of illness, years, mean (SD)	16.5 (2.2)	16.2 (2.5)
Duration of illness, years, mean (SD)	0.1 (0.3)	0.1 (0.4)
Prior number of hospitalizations, %		
None	46.6	48.0
1	26.4	25.5
2	14.4	9.2
≥ 3	12.5	17.3
PANSS total score, mean (SD)		
Double-blind Baseline	94.6 (11.5)	93.1 (10.8)
Extension phase Baseline	72.4 (17.7)	79.2 (18.1)
CGI-Severity, mean (SD)		
Double-blind baseline	4.9 (0.6)	4.8 (0.6)
Extension phase baseline	3.7 (1.0)	4.2 (1.0)

The following concomitant medications were used during the course of the pooled extension studies: anxiolytics (71/306; 23.2%), benzatropine or other anticholinergic medication (28/306; 9.2%), propranolol (6/306; 2.0%), and sedative/hypnotics (21/306; 6.9%).

A total of 208 patients (68.0%) completed 12 months of treatment with lurasidone, 68.3% in the core lurasidone group and 67.3% in the core placebo group. The Kaplan‒Meier estimates of the median [95% CI] time to discontinuation during the extension phase were similar in both groups: 375 [375, –] days vs. 371 [368, –] days, respectively. The Kaplan‒Meier estimate of the probability of discontinuation was 0.30 for both groups. The reasons for discontinuation in the extension phase (by original core lurasidone vs. placebo assignment) were as follows: lack of efficacy (5.3% vs. 3.1%), adverse events (7.7% vs. 9.2%), withdrawal of consent (9.6% vs. 13.3%), loss to follow-up (2.9% vs. 2.0%), and miscellaneous reasons (6.2% vs. 5.1%).

### Effectiveness

For the PANSS total score, the mean (95% CI) observed changes from the extension phase baseline for the overall safety sample were − 9.5 (− 11.0, − 8.0) at Month 3, − 11.8 (− 13.5, − 10.0) at Month 6, − 13.7 (− 15.6, − 11.8) at Month 9, − 15.3 (− 17.3, − 13.3) at Month 12, and − 10.5 (− 12.6, − 8.5) at the LOCF endpoint. For the subgroup treated with lurasidone in both the core and extension phases, the mean (95% CI) change from the core baseline was – 22.2 (− 24.3, − 20.0) at the acute endpoint (Week 6), and the mean (95% CI) changes from the extension phase baseline were − 7.5 (− 9.1, − 5.9) at Month 3, − 9.9 (− 11.8, − 8.0) at Month 6, − 12.0 (− 14.1, − 9.8) at Month 9, − 13.8 (− 16.1, − 11.4) at Month 12, and − 8.8 (− 11.2, − 6.3) at the LOCF endpoint.

**Fig. 1:**
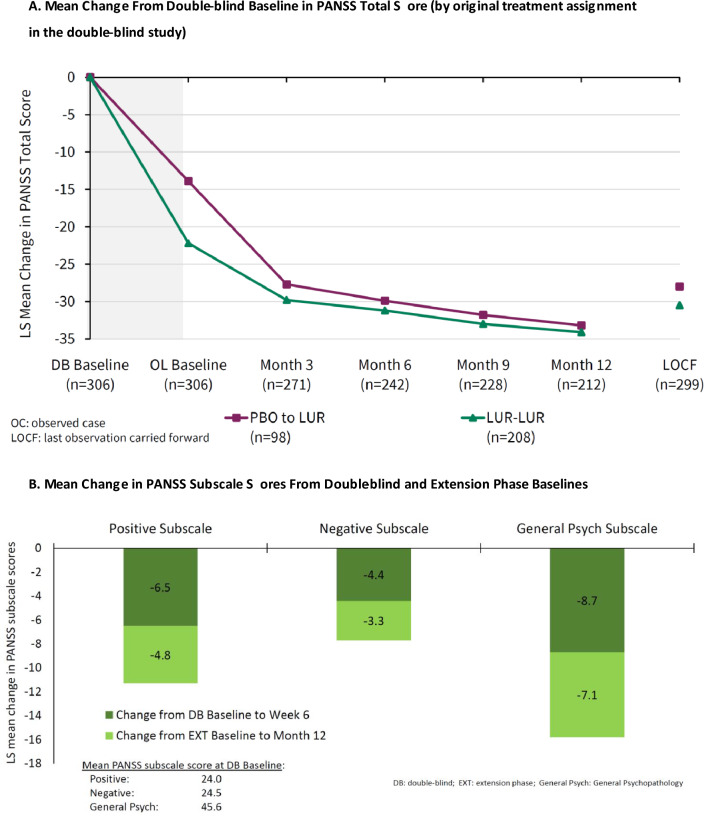
Effectiveness Measures. **A** Mean change in the PANSS total score from the double-blind baseline (by original treatment assignment in the double-blind study). **B** Mean Changes in PANSS Subscale Scores from Double-blind and Extension Phase Baselines

Figure 1A shows the mean change from the core baseline in the extension study (by original core lurasidone vs. placebo assignment). For patients switching from core placebo to lurasidone, the reduction in the PANSS total score was approximately “caught up” by Month 3 of the extension phase, with the improvement achieved in the core lurasidone group. For both groups combined, − 17.3/− 33.8 (51.2%) of the reduction in the PANSS total score (from core-baseline to month 12) occurred during the 12-month extension phase. For the subgroup treated with lurasidone in both the core and extension phases, − 17.3/− 33.8 (51.2%) of the reduction in the PANSS total score (from core-baseline to month 12) occurred during the 12-month extension phase.

The proportion of improvement from core-baseline to month 12 that occurred during the extension phase was 42.5% for the PANSS-positive subscale score, 42.9% for the negative subscale score, and 44.9% for the general psychopathology score (Fig. 1B).

**Fig. 2:**
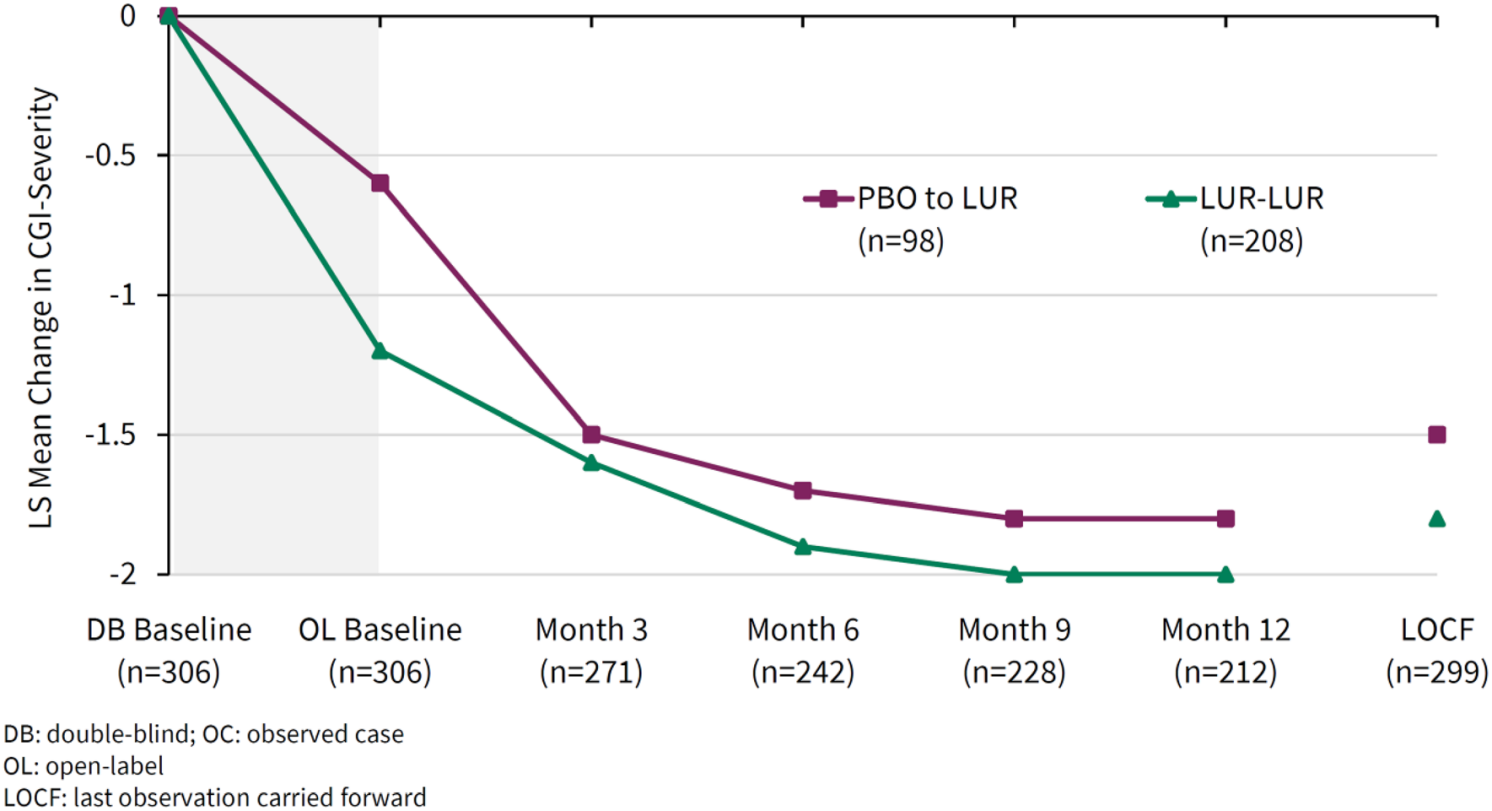
Mean change from double-blind baseline in CGI severity score (by original treatment assignment in the double-blind study)

On the CGI-Severity score, the mean (95% CI) observed changes from the extension baseline for the overall safety sample were − 0.6 (− 0.7, − 0.5) at Month 3, − 0.8 (− 1.0, − 0.7) at Month 6, − 1.0 (− 1.1,− 0.8) at Month 9, − 1.0 (− 1.2, − 0.9) at Month 12, and − 0.7 (− 0.8, − 0.6) at the LOCF endpoint. Figure 2 shows the mean change from the core baseline in the extension study (by original core lurasidone vs. placebo assignment). For patients who switched from core placebo to lurasidone, this reduction in the global CGI-S score approached that of Month 3 but never quite “caught up”, with the improvement achieved in the core lurasidone group.

**Fig. 3:**
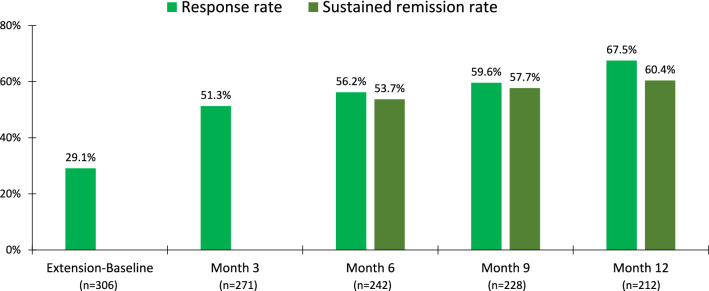
Response Rates (During 12 Months of Extension Phase Treatment) and Sustained Remission Rates (During 6 Months of Extension Phase Treatment)

For the overall safety sample, treatment response rates, based on a stringent reduction of ≥ 30% in the PANSS total score, showed an incremental increase during the 12-month course of treatment with lurasidone (Fig. 3). were 94.1% and 92.5% at week 26 (observed case) and 74.0% and 73.1% at the LOCF endpoint, respectively, and the response rates (≥ 50% reduction in PANSS) were 76.5% and 67.9% at week 26 (observed case) and 54.5% and 48.7% at the LOCF endpoint, respectively. The remission rates, based on the Andreasen criteria (**36**) and requiring 6 months of continuous symptom reduction, were 53.7% at Month 6, 57.7% at Month 9, and 60.4% at Month 12 (OC).

Among the 139 patients who met the responder criteria at Month 3, the Kaplan–Meier estimate of the probability of relapse at the end of the 12-month extension treatment period was 0.08. Among the 130 patients who met the Andreasen 6-month remission criteria, the Kaplan–Meier estimate of the probability of relapse at the end of the 12-month extension treatment period was 0.10.

### Safety

In the extension study, the overall incidence of AEs was 71.6%, resulting in an 8.2% discontinuation rate for AEs among all extension-phase patients. Twelve AEs were reported with an incidence ≥ 5%: headache (17.6%), anxiety (11.4%), schizophrenia (9.8%), nausea (9.8%), agitation (7.5%), agitation (7.2%), somnolence (6.9%), insomnia (5.9%), dizziness (5.6%), vomiting (5.6%), nasopharyngitis (5.2%), and weight increase (5.2%). Only one AE (weight increase) was meaningfully greater (≥ 5%) in the core placebo group than in the core lurasidone group (9.2% vs. 3.4%).

The mean (SD) changes from the core baseline in weight and BMI during 12 months of extension treatment with lurasidone were + 2.5 (5.1) kg and + 0.5 (1.6) kg/m^2^, respectively.

**Fig. 4:**
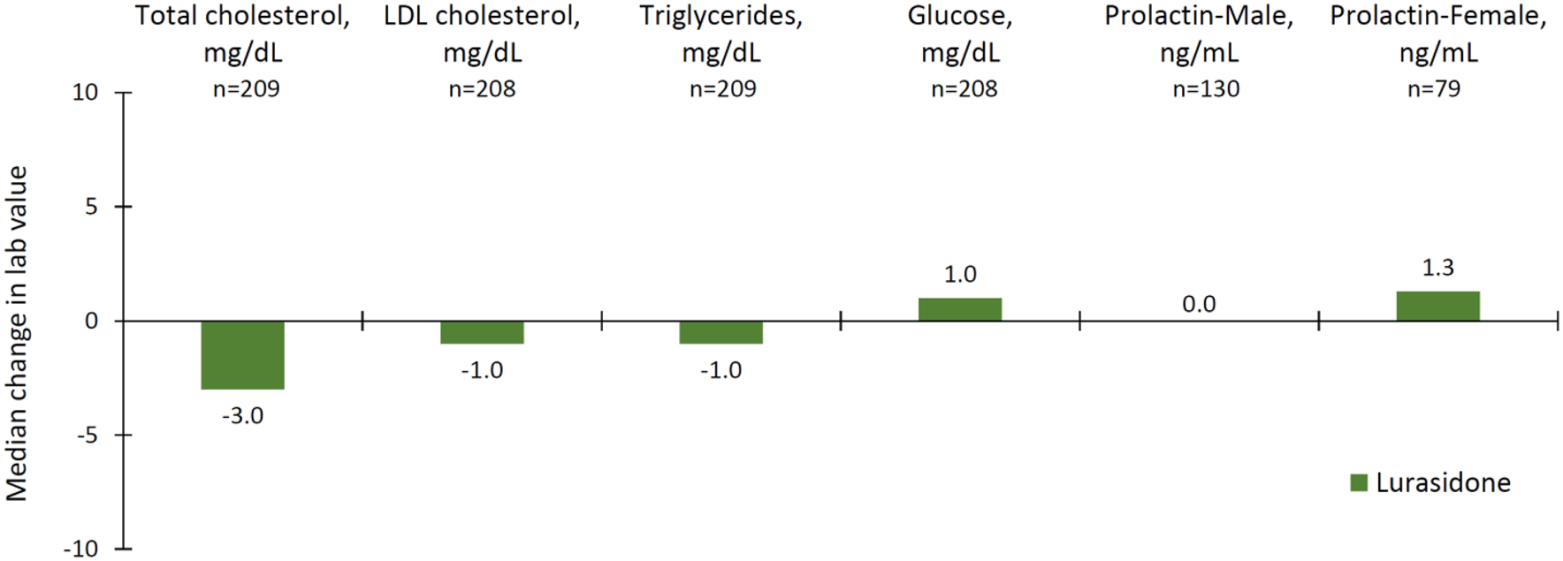
Key laboratory values: median change from double-blind baseline to month 12

The effect of 12 months of treatment with lurasidone revealed small median changes in metabolic laboratory parameters, including total cholesterol (− 3.0 mg/dL), LDL cholesterol (− 1.0 mg/dL), triglycerides (− 1.0 mg/dL), and glucose (+ 1.0 mg/dL), from the core baseline (Fig. 4). Treatment with lurasidone had minimal effect on median serum prolactin levels in both females (+ 1.3 ng/mL) and males (0.0 ng/mL).

## Discussion

In adolescents and young adults with schizophrenia, 12 months of lurasidone, in a dose range of 40–160 mg/day, was found to be an effective therapy. Long-term treatment was associated with a notable continued reduction in symptoms of schizophrenia across the PANSS-positive, negative, and general psychopathology subscales. Consistent with this, rates of treatment response (utilizing a ≥ 30% PANSS improvement criterion) increased incrementally from 29.1% at OL-Baseline to 67.5% at Month 12.

An intriguing finding of the current analysis of adolescents and young adults was that a greater proportion of improvement in the PANSS total score occurred later in treatment, during the extension phase, than is typically reported for adult treatment studies of schizophrenia. This can be seen by comparing the current results to a previously reported 12-month lurasidone treatment study [24] in adult patients with schizophrenia (mean age, 37 years; age of onset, 11 years; duration of illness, 11 years; baseline PANSS total score, 97.7). In that adult study, the initial 6 weeks of treatment with lurasidone was associated a 34.6 reduction in PANSS total score, while a smaller (5.0) reduction in PANSS total score occurred during an additional 12 months of treatment. In the present study, in contrast, in the subgroup treated with lurasidone in both the core and extension phases, the proportion of the reduction in the PANSS total score that occurred during the acute phase was notably smaller in both the OC analysis and the LOCF endpoint analysis. Further research is needed to confirm whether this is a replicable finding and, if so, what might account for the delayed response time.

In the present study, the remission rates based on the RSWG criteria, which require that a threshold of improvement be maintained for at least 6 months, [33] were 53.7% at Month 6 and 60.4% at Month 12. Remission is correlated with notably more favorable functional and occupational outcomes, as much as fourfold greater in first-episode schizophrenia, [34–36] and thus is an important treatment goal. Achieving remission is especially important because it is associated with a marked reduction in the risk of relapse. In the current younger patient population, a Kaplan‒Meier estimate of the probability of relapse was 8% in patients who met the responder criteria (≥ 30% improvement in the PANSS toral score); however, achieving SR (for 6 months) provided no additional relapse prevention benefit, at least not when utilizing the Andreasen criteria [33].

The relatively high rate of remission (and stringent response) and the associated low 12-month rate of relapse are notable given that all patients in the current 12-month study had just experienced an acute psychotic episode with a mean PANSS total score of 94 and had not completed a 3-month stabilization period that is frequently included in remission analyses. The relapse rate at 12 months of 8% in the current study compares favorably to the estimated relapse rate at 1 year of 27% reported in a meta-analysis of adult clinical trials in schizophrenia patients (N = 65 studies; N = 6,493 patients) [37]. We are not aware of similar relapse data based on clinical trials in adolescents and young adults.

Twelve months of treatment with lurasidone in this younger cohort was generally well tolerated. AEs were typically mild-to-moderate in severity and resulted in study discontinuation in 8.2% of patients. Long-term treatment with lurasidone was associated with no clinically meaningful changes in movement disorder scales and with minimal effects on weight, metabolic parameters, and prolactin. These findings are consistent with results from previous short-term and long-term studies in both schizophrenia patients and bipolar disorder patients [38, 39]. The low risk of weight gain and metabolic abnormalities on lurasidone may be due, at least in part, to its lack of activity at 5HT2C and histamine H1 receptors [40–42].

Several study limitations should be mentioned. Most notably, this was a pooled post hoc analysis, and therefore, the results should be viewed as exploratory. Additionally, the absence of a placebo or active comparator antipsychotic reduces the ability to benchmark the current effectiveness results, especially in terms of remission and relapse.

In conclusion, the results of this post hoc analysis of adolescents and young adults with schizophrenia revealed that long-term treatment with lurasidone, at doses of 40–160 mg/day, was safe, well tolerated, and effective in maintaining improvement in schizophrenia symptoms and preventing relapse while having minimal effects on weight, metabolic parameters, and prolactin.

## Data Availability

The data from the post-hoc analyses summarized in this article are available upon reasonable request from the corresponding author.
